# Sporadic Occurrence of Ensitrelvir-Resistant SARS-CoV-2, Japan

**DOI:** 10.3201/eid3006.240023

**Published:** 2024-06

**Authors:** Akihiro Doi, Masayuki Ota, Masumichi Saito, Shutoku Matsuyama

**Affiliations:** National Institute of Infectious Diseases, Tokyo, Japan

**Keywords:** COVID-19, ensitrelvir, SARS-CoV-2, severe acute respiratory syndrome coronavirus 2, SARS, coronavirus disease, coronavirus, GISAID, antimicrobial resistance, respiratory infections, viruses, zoonoses, Japan

## Abstract

Using the GISAID EpiCoV database, we identified 256 COVID-19 patients in Japan during March 31–December 31, 2023, who had mutations in the SARS-CoV-2 nonstructural protein 5 conferring ensitrelvir resistance. Ongoing genomic surveillance is required to monitor emergence of SARS-CoV-2 mutations that are resistant to anticoronaviral drugs.

Ensitrelvir fumaric acid (hereafter ensitrelvir) is a drug that inhibits the 3-chymotrypsin-like protease of SARS-CoV-2, also known as nonstructural protein 5 (NSP5), thereby inhibiting virus replication (*1*–*3*). Ensitrelvir was first approved for use in Japan on November 22, 2022. After drug approval, ensitrelvir was prescribed widely after March 2023 by many internal medicine clinics throughout Japan for COVID-19 treatment; indeed, 227,216 doses have been distributed in Japan since March 31, 2023 (*4*). However, in other countries, ensitrelvir prescriptions have been limited to clinical trials. To track emergence of SARS-CoV-2 mutations conferring resistance to ensitrelvir, we searched the GISAID EpiCoV database (https://www.gisaid.org), which contains virus genome sequences collected from COVID-19 patients worldwide.

We counted the number of SARS-CoV-2 cases that had NSP5 amino acid substitutions conferring ensitrelvir resistance (*5*–*8*) from March 31, 2023, the date ensitrelvir was first prescribed by general internal medicine clinics, through December 31, 2023 (Table). Although the occurrence of some NSP5 amino acid substitutions showed a regional bias, most were not associated with ensitrelvir prescription. For example, of the 77 sequences harboring the M46I amino acid substitution in NSP5 observed in the United States, 66 were identified in specimens collected during the same period in May 2023, suggesting an association with a cluster that likely arose from a sporadic occurrence. However, the M49L amino acid substitution in NSP5, which confers ensitrelvir resistance without attenuating virus infection both in vitro and in vivo (*5*), was observed in 256/49,414 (0.55%) virus sequences from Japan. By comparison, the M49L substitution was observed in 277/845,796 (0.03%) virus sequences deposited globally in the GISAID database; therefore, 92.4% of the deposited M49L mutant sequences of NSP5 were from Japan. The M49L substitution is caused by transversion of adenine at position 10199 within the SARS-CoV-2 NSP5 coding sequence to either cytosine or uracil. Of the 277 sequences with the M49L amino acid substitution, 89 (32.1%) had g.10199A>C, and 188 (67.9%) had g.10199A>U nucleotide mutations. Only 2 sequences had g.10199A>G transitions despite transitions generally occurring more frequently than transversions, which indicates ensitrelvir exerts high selective pressure on SARS-CoV-2 in COVID-19 patients. The number of virus sequences with M49L substitutions began to increase in June, peaked in September, and then decreased in November of 2023, a pattern corresponding to the number of COVID-19 cases observed throughout Japan during that period (Figure). In Japan, the monthly occurrence rate of ensitrelvir-resistant SARS-CoV-2 infections was significantly higher during the 9 months after initiating widespread ensitrelvir prescriptions than during the preceding period (Appendix Figure 1).

**Table:** Number of mutations in NSP5 causing ensitrelvir resistance during March 31–December 31, 2023, in study of sporadic occurrence of ensitrelvir-resistant SARS-CoV-2, Japan*

Amino acid substitutions‡	No. cases from GISAID database†					
	Globally	Japan	China	United States	Europe	Others
T45I	25	0	1	4	15 (60.0)	5
D48Y	0	0	0	0	0	0
M49I	90	6	0	77 (85.6)	5	2
M49L	277	256 (92.4)	2	15	0	4
M49T	1	0	0	0	0	1
M49V	1	0	0	1	0	0
L50F	85	6	2	31	26	20
P52L	4	2	0	0	0	2
Y54C	0	0	0	0	0	0
S144A	4	2	0	0	1	1
E166A	1	1	0	0	0	0
E166V	23	0	1	4	10	8
L167F	0	0	0	0	0	0
P168del	4	0	0	0	0	4
A173T	23	0	1	6	11	5
A173V	23	2	0	6	3	12 (52.2)
Q192R	2	0	0	0	1	1

*Numbers in parentheses are percentages of total global cases (if a particular mutation was found in >10 cases and was responsible for >50% of global cases). del, deletion; NSP5, nonstructural protein 5. †Sequences were extracted from the GISAID EpiCoV database (https://www.gisaid.org). ‡Amino acid substitutions in NSP5 that trigger emergence of ensitrelvir-resistant SARS-CoV-2 (*5**–**8*).

**Figure:**
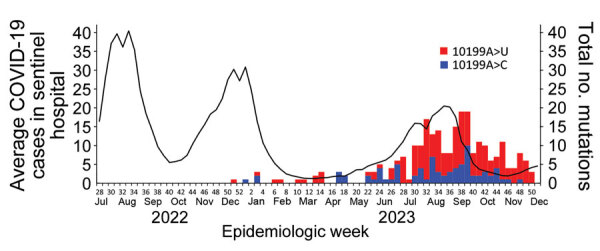
Sporadic occurrence of ensitrelvir-resistant SARS-CoV-2 mutants during December 2022–December 2023 in Japan. Solid line indicates the average number of COVID-19 cases. Weekly numbers of SARS-CoV-2 sequences harboring g.10199A>U and g.10199A>C mutations within nonstructural protein 5 were extracted from the GISAID EpiCoV database (https://www.gisaid.org). Number of mutations were aligned on the same time axis as the weekly average number of COVID-19 patients identified at ≈5,000 sentinel hospitals organized by the Japan Ministry of Health, Labor and Welfare. Scales for the y-axes differ substantially to underscore patterns but do not permit direct comparisons.

We constructed a phylogenetic tree as described previously (*9*). We downloaded whole-genome sequences from 277 SARS-CoV-2 mutants collected globally during March 31–December 31, 2023, and constructed the tree by using Nextstrain (https://www.nextstrain.org) and 570 reference genomes (Appendix Figure 2). Single sporadic occurrences of ensitrelvir-resistant mutants that were not linked to each other in the phylogenetic tree were counted if only 1 case occurred in a clade or if 1 case occurred >2 segments downstream of different branches from other cases belonging to the same clade, as described previously (*9*). Sporadic occurrence of g.10199A>C was detected 24 times and g.10199A>U was detected 22 times.

Although SARS-CoV-2 g.10199A>C and g.10199A>U mutations were detected nationwide in Japan, they were more frequent in populated metropolitan areas (Appendix Figure 3). Sporadic occurrence of mutants not linked to human-to-human virus transmission within a prefecture was defined as detection of 1 genome with either the g.10199A>C or g.10199A>U mutation or defined as detection of 1 mutant genome collected >1 month apart from others. We considered >105 genome mutations, 46 with g.10199A>C and 59 with g.10199A>U, to be sporadic occurrences (Appendix Figure 3), suggesting that ensitrelvir-resistant SARS-CoV-2 emerges frequently in Japan.

In conclusion, COVID-19 patients in Japan are usually prescribed ensitrelvir immediately after receiving positive results from a rapid immunochromatographic SARS-CoV-2 test. The Japan Ministry of Health, Labour and Welfare has conducted surveillance by using next-generation sequencing to enable rapid detection of drug-resistant SARS-CoV-2 (*10*). We examined the occurrence of ensitrelvir-resistant SARS-CoV-2 after widespread ensitrelvir prescription in Japan. Replication of those ensitrelvir-resistant mutant viruses in individual patients is thought to be driven predominantly by selective pressure exerted by the drug, leading to sporadic occurrence. The decreased occurrence of ensitrelvir-resistant SARS-CoV-2 after October 1, 2023, might be because patients are required to pay a portion of their medical costs, which could thereby decrease the number of ensitrelvir prescriptions. Increasing use of ensitrelvir worldwide will likely increase the frequency of mutations in SARS-CoV-2 causing ensitrelvir resistance. Ongoing genome surveillance using next-generation sequencing is required to monitor emergence of SARS-CoV-2 mutants that are resistant to anticoronaviral drugs.

Appendix Additional information for sporadic occurrence of ensitrelvir-resistant SARS-CoV-2, Japan.
