# Supplementary material for: Sporadic Occurrence of Ensitrelvir-Resistant SARS-CoV-2, Japan
Source: Emerg Infect Dis. 2024 Jun;30(6):1289–91. doi: 10.3201/eid3006.240023 (PMC11138985; doi:10.3201/eid3006.240023)
Supplement: Appendix — Additional information for sporadic occurrence of ensitrelvir-resistant SARS-CoV-2, Japan. [file 24-0023-Techapp-s1.pdf]

Article DOI: https://doi.org/10.3201/eid3006.240023

EID cannot ensure accessibility for supplementary materials supplied by authors. Readers who have difficulty accessing supplementary content should contact the authors for assistance.

Sporadic Occurrence of Ensitrelvir-Resistant SARS-CoV-2, Japan

Appendix

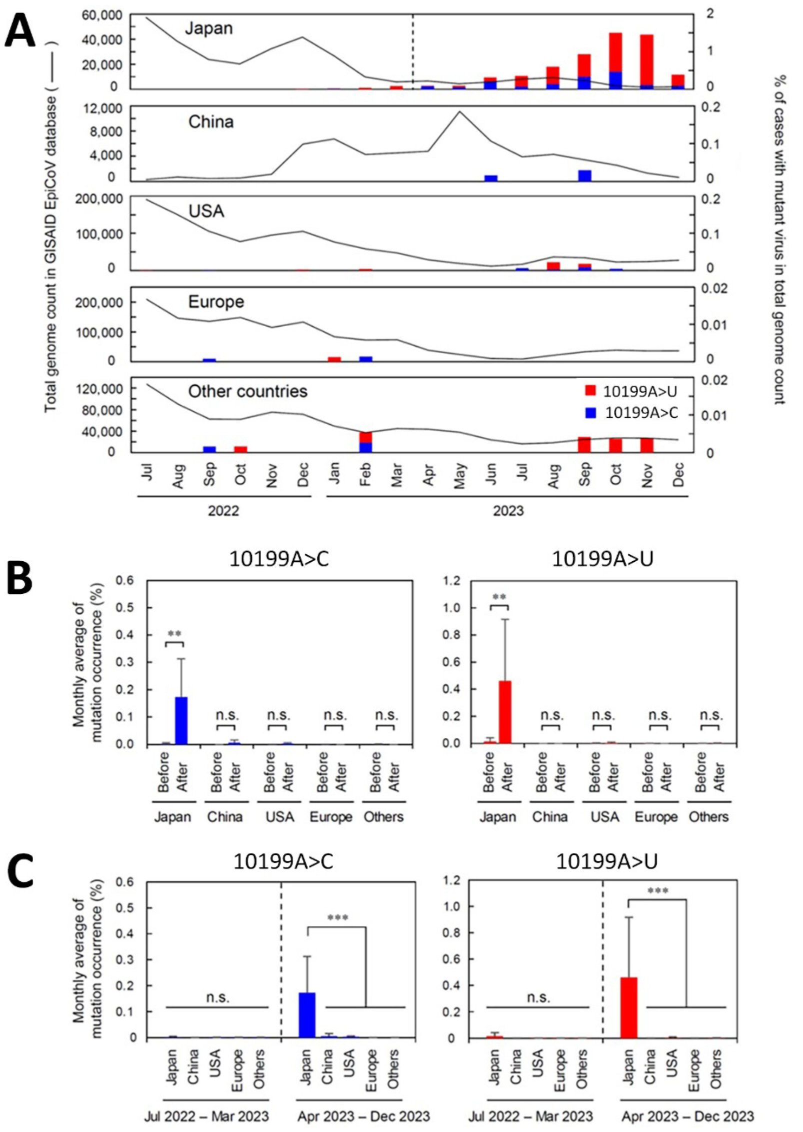

Appendix Figure 1. Occurrence of ensitrelvir-resistant SARS-CoV-2 in different countries. Data are from the GISAID EpiCoV database (https://www.gisaid.org). A) Number of mutations conferring ensitrelvir resistance reported each month during July 2022–December 2023. Monthly counts of SARS-CoV-2 sequences harboring the g.10199A>C and g.10199A>U mutations were divided by the total genome count deposited in the database for each country/region. Dotted line indicates date of widespread initiation of ensitrelvir prescription in internal medicine clinics in Japan. B) Monthly comparisons of percentages of SARS-CoV-2 sequences harboring the g.10199A>C and g.10199A>U mutations for each country/region before (July 2022–March 2023) and after (April 2023–December 2023) widespread initiation of ensitrelvir prescription in Japan. Comparisons were made by using t-tests. Error bars indicate SD. **, p<0.01. C) Monthly comparisons of SARS-CoV-2 sequences harboring the g.10199A>C and g.10199A>U mutations between countries/regions. Comparisons were made by using 1-way analysis of variance. Error bars indicate SD. n.s., not significant; ***, p<0.001.

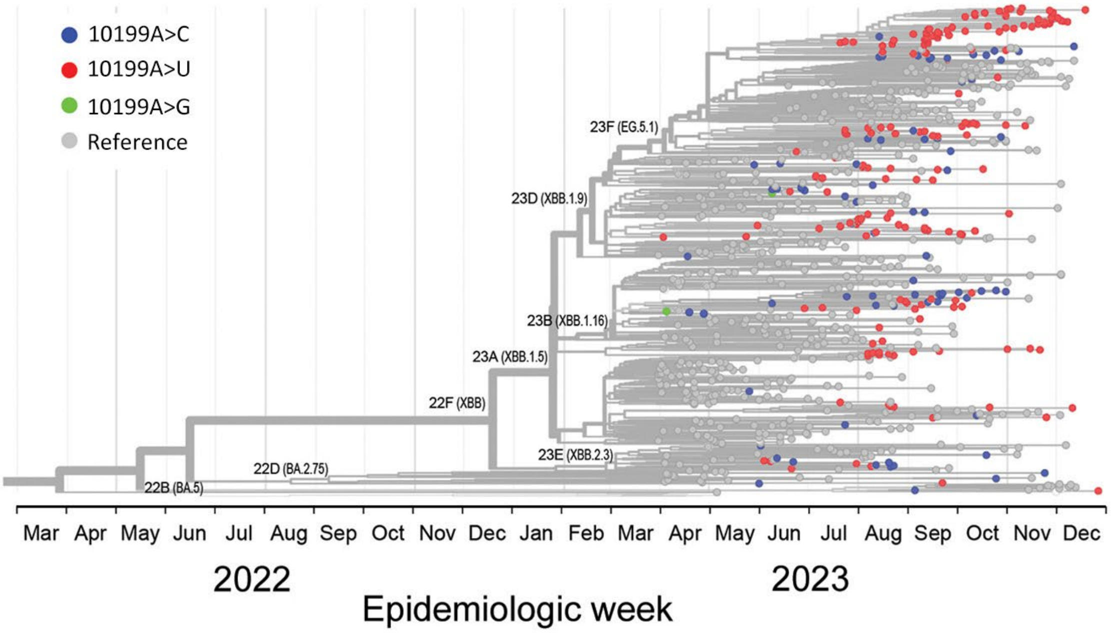

Appendix Figure 2. Time-scaled phylogenetic analysis of ensitrelvir-resistant SARS-CoV-2 mutants. Genomic sequences were deposited globally into the GISAID EpiCoV database (https://www.gisaid.org). SARS-CoV-2 variants are indicated at different branches of the tree. Phylogenetic tree was constructed for g.A10199A>C, g.10199A>U, and g.10199A>G SARS-CoV-2 mutants and 570 background reference sequences obtained from Nextstrain (https://www.nextstrain.org) by using the workflow provided by the Nextstrain command-line interface, as previously described (*9*).

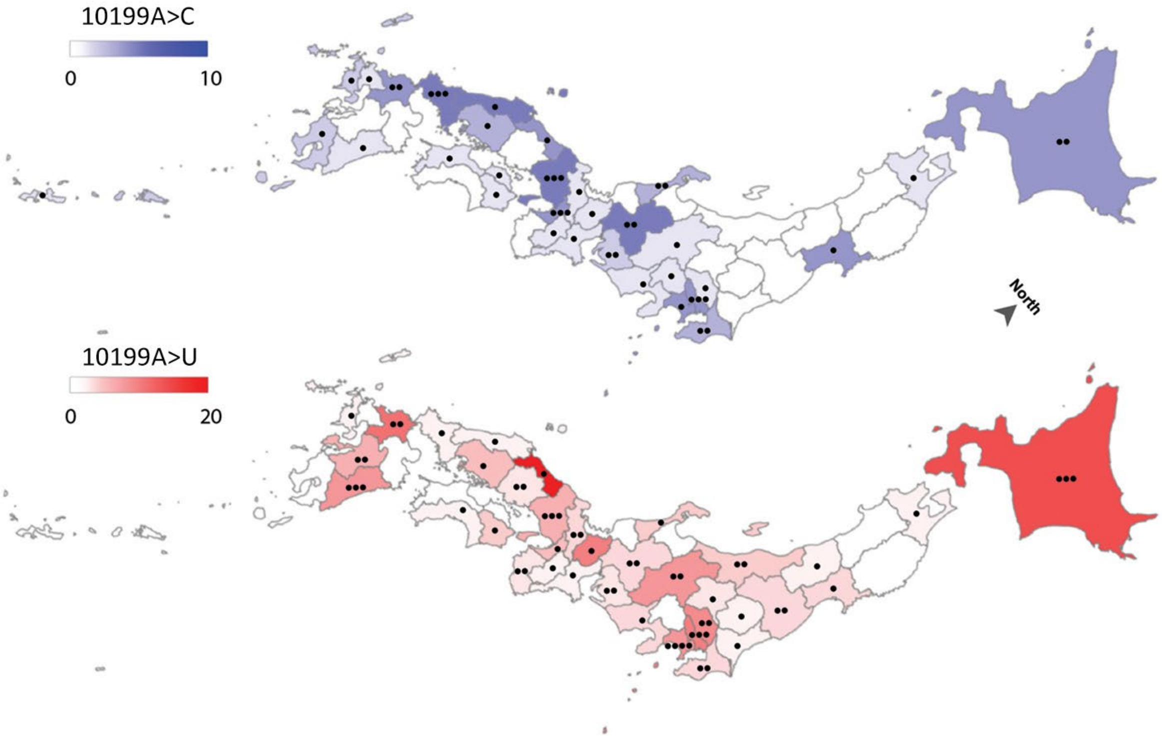

Appendix Figure 3. Geographic distribution of patients in Japan infected with SARS-CoV-2 carrying the g.10199A>C or g.10199A>U mutations in nonstructural protein 5. In each prefecture, 1 dot indicates 1 sporadic occurrence not linked to other cases.
